# Yezo Virus Infection in Tick-Bitten Patient and Ticks, Northeastern China

**DOI:** 10.3201/eid2904.220885

**Published:** 2023-04

**Authors:** Xiaolong Lv, Ziyan Liu, Liang Li, Wenbo Xu, Yongxu Yuan, Xiaojie Liang, Li Zhang, Zhengkai Wei, Liyan Sui, Yinghua Zhao, Zhijun Hou, Feng Wei, Shuzhen Han, Quan Liu, Zedong Wang

**Affiliations:** First Hospital of Jilin University, Changchun, China (Z. Liu, W. Xu, L. Sui, Y. Zhao, Q. Liu, Z. Wang);; Inner Mongolia General Forestry Hospital, Yakeshi, China (X. Lv, S. Han);; Jilin Agricultural University, Changchun (Z. Liu, Y. Yuan, X. Liang, F. Wei);; Chinese Academy of Agricultural Sciences, Changchun (L. Li, L. Zhang, Q. Liu, Z. Wang);; Foshan University, Foshan, China (Z. Wei, Q. Liu);; Northeast Forestry University, Harbin, China (Z. Hou)

**Keywords:** Yezo virus, viruses, infection, tick-bitten patient, ticks, Ixodes persulcatus, vector-borne infections, zoonoses, northeastern China

## Abstract

We identified Yezo virus infection in a febrile patient who had a tick bite in northeastern China, where 0.5% of *Ixodes persulcatus* ticks were positive for viral RNA. Clinicians should be aware of this potential health threat and include this emerging virus in the differential diagnosis for tick-bitten patients in this region.

Tickborne orthonairoviruses have been considered a major public health threat worldwide (*1*). In China, other than Crimean-Congo hemorrhagic fever virus, there are 3 emerging orthonairoviruses: Tacheng tick virus 1 (*2*), Songling virus (*3*), and Beiji nairovirus (*4*). Those viruses have been associated with human febrile illness in northeastern and northwestern China.

Yezo virus (YEZV), a new tickborne orthonairovirus discovered in Japan in 2021, can cause acute febrile illness in humans, whose clinical symptoms include thrombocytopenia and leukopenia (*5*). We report a case of YEZV infection in a tick-bitten patient and provide molecular evidence of YEZV infection in ticks in northeastern China.

## The Study

The research protocol was approved by the human bioethics committee of Inner Mongolia General Forestry Hospital and the First Hospital of Jilin University, China. During 2018–2020, a total of 402 blood samples from tick-bitten patients were collected at the Inner Mongolia General Forestry Hospital (164 in 2018, 97 in 2019, and 141 in 2020) for viral detection by using reverse transcription PCR (Appendix Table 1). Results showed that 1 sample collected in 2018 was YEZV positive.

**Figure 1:**
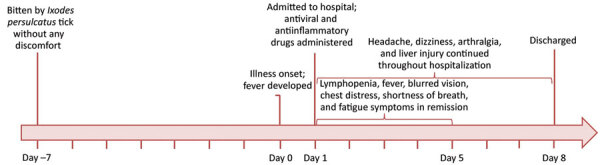
Timeline of the clinical course for a tick-bitten person infected with Yezo virus, northeastern China.

The patient was a 33-year-old man who lived on a farm in the Oroqen Autonomous Banner of Hulunbuir, Inner Mongolia, northeastern China, who had no history of underlying diseases. On June 18, 2018, he noticed a tick embedded on his back after he grazed horses on a mountain. The tick was removed intact by using tweezers in a local clinic and identified as *Ixodes persulcatus*. At that time, no obvious clinical symptoms, such as rash, itching, and discomfort, occurred. However, fever developed, followed by light headache, dizziness, blurred vision, chest distress, shortness of breath, fatigue, and arthralgia in 1 week (Table 1; Figure 1). No gastrointestinal (e.g., nausea, vomit, diarrhea) or hemorrhagic (e.g., melena, petechia, and ecchymosis) symptoms occurred.

**Table 1:** Characteristics of a tick-bitten patient infected with Yezo virus, northeastern China*

Characteristic	Result and treatment
Fever	
Temperature at admission, °C	39.5
Highest temperature, °C	39.6
Complications	
Headache	Yes
Dizziness	Yes
Blurred vision	Yes
Arthralgia	Yes
Chest distress	Yes
Shortness of breath	Yes
Fatigue	Yes
Bacterial co-infection	No
Tickborne pathogen detection	
TBEV	Negative
SFTSV	Negative
Alongshan virus	Negative
Songling virus	Negative
Beiji nairovirus	Negative
Yezo virus	Positive
*Borrelia* spp.	Negative
*Rickettsia* spp.	Negative
*Anaplasma* spp.	Negative
*Babesia* spp.	Negative
Treatment	
Day 1–4	Ribavirin (1 g IV), azithromycin (0.5 g IV), rocephin (3 g IV) daily
Day 1–8	Glycyrrhizin (3 tablets orally daily)

*IV, intravenous; SFTSV, severe fever with thrombocytopenia syndrome virus; TBEV, tick-borne encephalitis virus.

Laboratory tests identified lymphocytopenia and neutrophilia, which accounted for 15.0% and 75.9% of the total leukocyte count, respectively. However, leukocyte counts (4,460 cells/mL) and platelet counts (199,000 cells/mL) were within reference ranges. Serum levels of liver aminotransferases were slightly increased (alanine aminotransferase 40 U/L, aspartate aminotransferase 44 U/L, and γ-glutamyltransferase 124 U/L) (Appendix Table 2). The serum level of C-reactive protein increased to 11.1 mg/L.

The patient was hospitalized for 8 days. Headache, dizziness, and arthralgia continued throughout the hospitalization, whereas the clinical signs of fever, blurred vision, chest distress, shortness of breath, and fatigue were relieved or disappeared (Figure 1). Lymphopenia continued until day 5, but counts of leukocytes, platelets, and erythrocytes and the hemoglobin level were all within reference ranges. Evidence of liver damage continued through the discharge date (alanine aminotransferase 56 U/L and γ -glutamyltransferase 181 U/L).

A blood sample was collected at admission and was negative for the tick-borne pathogens that have been identified in northeastern China (Table 1). The patient was empirically given ribavirin (1 g), azithromycin (0.5 g), and rocephin (3 g) intravenously each day during the first 4 days of hospitalization (Table 1). Glycyrrhizin was used to treat liver injury. The patient was discharged on the 8th day of hospitalization, although some clinical manifestations, such as headache, dizziness, and arthralgia, were still present. Two weeks later, the patient had recovered completely.

During April 2020‒July 2021, a total of 2,830 ticks were collected from Heilongjiang, Jilin, and Inner Mongolia in northeastern China (214 *Haemaphysalis japonica*, 431 *H. concinna*, 1,110 *Dermacentor silvarum*, and 1,075 *I. persulcatus*) (Table 2). YEZV RNA was detected in *I. persulcatus* ticks by using reverse transcription PCR; overall prevalence was 0.5% (95% CI 0.2%–1.0%) (Table 2). The prevalence of YZEV infection in *I. persulcatus* ticks in Inner Mongolia, Heilongjiang, and Jilin varied from 0.4% to 0.5%. No positive sample was detected in other tick species.

**Table 2:** Detection of Yezo virus RNA in *Ixodes persulcatus* ticks, northeastern China*

Province	Sampling time	No. pools/no. ticks	No. positive pools	Positive rate (95% CI)
Heilongjiang	2021	63/625	3	0.5 (0.1–1.3)
Jilin	2020–2021	19/192	1	0.5 (0.0–2.5)
Inner Mongolia	2021	26/258	1	0.4 (0.0–1.9)
Total	2020–2021	108/1,075	5	0.5 (0.2–1.0)

*Prevalence of Yezo virus infection in ticks was calculated by using PooledInfRate Excel Add-In version 4.0 (Microsoft, https://www.microsoft.com) and a 1-sample analysis with a bias-corrected maximum likelihood estimation method with 95% CIs and a scale of 100.

We obtained complete genomes of 6 YEZV strains (1 from the patient and 5 from *I. persulcatus* ticks) by using specific primers (Appendix Table 1). YEZV has a genomic structure of typical orthonairoviruses (*5*). The complete genome of YEZV identified in this study included large (12,122-nt), medium (4,256-nt) and small (1,697-nt) segments (Appendix Table 3), which encoded a 3,938-aa large protein, a 1,356-aa glycoprotein precursor, and a 502-aa nucleocapsid (Appendix Figure 1). YEZV strain H-IM01 from the patient showed high sequence identities with those detected in ticks (T-HLJ01–03, T-JL01, and T-IM01) and nucleotide identities of 99.6%–100% (Appendix Tables 4, 5). Strains isolated from northeastern China were clustered with strains detected in tick-bitten patients in Japan and showed high nucleotide identities of 97.2%–98.8% (Appendix Tables 4, 5).

**Figure 2:**
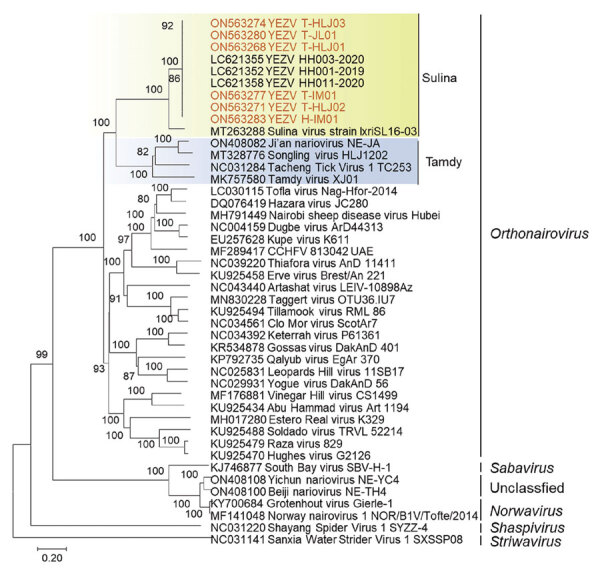
Phylogenetic analyses of Yezo virus from a tick-bitten person and *Ixodes persulcatus* ticks, northeastern China (red text), and references viruses. Sequences of representative viral strains were downloaded from National Center for Biotechnology Information public databases (https://www.ncbi.nlm.nih.gov) and aligned together using MEGA version 7.0 (https://www.megasoftware.net). A bootstrapping analysis of 1,000 replicates were conducted, and values >70 were considered significant and are shown. Shading indicates Sulin virus genogroup strains (green) and Tamdy virus strain (blue). Numbers along branches are bootstrap values. Scale bar indicates amino acid substitutions per site.

The YEZV strains from China were genetically related to Sulina virus discovered in *I. ricinus* ticks in Romania (*6*), showing complete genome nucleotide identities of 59.7%–70.3% and large protein amino acid identities of 82.3%–82.5%; they were grouped into the genogroup Sulina (Figure 2; Appendix Tables 4, 5, Figure 2) (*5*). Phylogenetic analysis indicated that viruses in the genogroup Sulina had a close relationship with Tamdy virus; those viruses showed nucleotide identities of ≈50% and large protein amino acid identities of ≈45% with each other. All viral genome sequences have been submitted to GenBank (Appendix Table 3).

## Conclusions

Clinical manifestations of the YEZV-infected patient in China were milder than those reported for patients in Japan, where leukopenia, lymphocytopenia, thrombocytopenia, coagulation disorder, and increased levels of liver and heart enzymes have been observed (*5*); only mild lymphocytopenia and mildly increased levels of liver enzymes were found in the patient in this study. The 2 patients in Japan were a 59-year-old man and a 41-year-old man who had medical histories of hyperuricemia and hyperlipidemia, and the patient from China was 33-year-old man who had no underlying disease. The YEZV-infected patient in this study was given ribavirin on days 1–4, but the patients in Japan were not given ribavirin. The clinical signs of YEZV infection might be related to age, medical history, and medication. No gastrointestinal symptoms, such as nausea, vomiting, and diarrhea, or hemorrhagic symptoms, such as melena, petechia, and ecchymosis, occurred in any of the YEZV-infected patients.

The infection rate of YEZV was low (1/402) in tick-bitten patients in northeastern China, compared with YEZV patients in Japan (5/248). To date, no severe YEZV-infected patient has been reported, and the patients in the 2 countries recovered completely. Thus, active surveillance should be performed on the tick-bitten populations to evaluate the prevalence and clinical characteristics of YEZV infection in the studied regions.

YEZV RNA has been detected in *H. megaspinosa*, *I. ovatus*, and *I. persulcatus* ticks in Hokkaido, Japan, showing a prevalence of 0.0%–5.7% (*5*). In this study, YEZV was detected only in *I. persulcatus* ticks, showing a prevalence of 0.5% (95% CI 0.2%–1.0%), and other tick species, such as *H. japonica*, *H. concinna*, and *D. silvarum*, were negative for YEZV RNA. Those results indicate that *I. persulcatus* ticks might serve as a potential vector for YEZV in northeastern China.

YZEV was identified in a tick-bitten patient who had febrile illness and *I. persulcatus* tick bites in northeastern China. Phylogenetic analysis confirmed the association between febrile illness and the virus. To date, there are >8 pathogenetic tickborne viruses in humans and animals found in northeastern China: tickborne encephalitis virus (*7*), severe fever with thrombocytopenia syndrome virus (*8*), Nairobi sheep disease virus (*9*), Alongshan virus (*10*), Jingmen tick virus (*11*), Songling virus (*3*), Beiji nairovirus (*4*), and YEZV. Differential diagnosis of these tickborne viruses should be conducted in for febrile patients who have a history of tick bites in northeastern China.

Appendix Additional information on Yezo virus infection in tick-bitten patient and ticks, northeastern China.
