# Supplementary material for: Yezo Virus Infection in Tick-Bitten Patient and Ticks, Northeastern China
Source: Emerg Infect Dis. 2023 Apr;29(4):797–800. doi: 10.3201/eid2904.220885 (PMC10045709; doi:10.3201/eid2904.220885)
Supplement: Appendix — Additional information on Yezo virus infection in tick-bitten patient and ticks, northeastern China. [file 22-0885-Techapp-s1.pdf]

Article DOI: https://doi.org/10.3201/eid2904.220885

EID cannot ensure accessibility for supplementary materials supplied by authors. Readers who have difficulty accessing supplementary content should contact the authors for assistance.

Yezo Virus Infection in Tick-Bitten Patient and Ticks, Northeastern China

Appendix

Appendix Table 1. Primers used in this study

Segment	Primer	Position (bp)*	Sequence (5′→3′)	Polarity	Amplicon (bp)
	Detection				
	YEZV F	1412	ACCCCACATACTCTCTAYAACGA	+	255
	YEZV R2	1646	TACTGTGGCCTTGATCTCACT	-	
	YEZV R1	1679	CTGAAGCCTCCCTAAGTGC	-	
	Genome amplification				
Large					
	1 YEZV F	1	ACATCTATCCTGCAATCCC	+	964
	1 YEZV R1	962	GGTGYCTYCCAACTTTCCC	-	
	1 YEZV R2	937	TGTTTGTTCGTTTACCAGC	-	
	2 YEZV F1	869	CTACTGTCCAAGCCAACCA	+	1218
	2 YEZV F2	900	GTCCTGTTCGTAGACTCCCAGC	+	
	2 YEZV R	2099	TCAAAMAGAACCTCGCACTC	-	
	3 YEZV F1	2017	AGTGCCCATAGCAATYATC	+	1066
	3 YEZV R2	3063	TCTGTCCATTGTAACYCCTT	-	
	3 YEZV R1	3272	CTGGCTATACCTTTGATTGCAT	-	
	4 YEZV F	2874	TCATGGTTGCCAACATCAGC	+	1029
	4 YEZV R2	3903	ATGAAGTTTAACCTCTTTTCGC	-	
	4 YEZV R1	3976	GTCAGACTTGTCCACTATA	-	
	5 YEZV F	3751	AGCCTGCAAGATAATGAACAC	+	926
	5 YEZV R2	4658	CCAGCCCAAACACAACTGA	-	
	5 YEZV R1	4836	ATACCAGCTGTAAGTAAAGCC	-	
	6 YEZV F	4597	GACAGGCAACTTTCTTGACC	+	1028
	6 YEZV R1	5786	TTTCTGTGCCTCTGTTAGTGT	-	
	6 YEZV R2	5605	TTGGCTCAGACACAATGGAT	-	
	7 YEZV F	5493	TCATTCAAGCYTTCACGGAG	+	1147
	7 YEZV R1	6684	CTCATCAGGCTTCATTGCAT	-	

Segment	Primer	Position (bp)*	Sequence (5′→3′)	Polarity	Amplicon (bp)
	7 YEZV R2	6619	CAGCAGTATCTGTATAGCCAA	-	
	8 YEZV F	6500	GCAWTGAAACGGCTTACTTC	+	1098
	8 YEZV R1	7764	TCTTACTTATTGCTCCGCTGA	-	
	8 YEZV R2	7577	GTTGTAACATGTCATCGCCAT	-	
	9 YEZV F1	7446	TGGCAAGCAAATACAAACTCG	+	1156
	9 YEZV F2	7526	GACCAGCCCATGATACAGT	+	
	9 YEZV R	8661	ATCTAACAAGGCCAAATGTCC	-	
	10 YEZV F	8559	AGGATTATGACGGATTCCCT	+	946
	10 YEZV R1	9577	GGCCTCCGTAATCAACCCTG	-	
	10 YEZV R2	9485	TTGACCTGCTCAGATGCATT	-	
	11 YEZV F	9377	ATGCAGTTTTCAGAGCCGAA	+	1174
	11 YEZV R1	10622	GCGGTTCACTACAGTCACA	-	
	11 YEZV R2	10531	GTGTAAAGCCCAACATCCTT	-	
	12 YEZV F	10450	CCTGCTTCCAACCTATCCAA	+	913
	12 YEZV R1	11371	AGACCTCTGTCATCTAGGCTR	-	
	12 YEZV R2	11344	CAGTTCGTTCTTGGCACTC	-	
	13 YEZV F1	10901	GAGCTAAGCAGCAAGTCACA	+	985
	13 YEZV F2	11137	CAGCAACATTGACGTCCARC	+	
	13 YEZV R	12101	GTTCCTGCATACCCCCCTATTA	-	
Medium					
	1 YEZV F	1	ACTTGCAGCGACCCCCCCAAAG	+	832
	1 YEZV R1	851	TCCACCACTGCCTTACAGCC	-	
	1 YEZV R2	813	ATAACCAGTWAGATAGGCAA	-	
	2 YEZV F	763	ATCAGARAATATAAAGGTCAGC	+	835
	2 YEZV R1	1631	GCTCGACAGACATAGATTGC	-	
	2 YEZV R2	1576	TAGATCAACCTTATTGGTGTGC	-	
	3 YEZV F	1518	CTGCTCCTAAAGGAAGTCTWG	+	836
	3 YEZV R1	2397	CCCTATTTTGACTTTGCCAAC	-	
	3 YEZV R2	2333	CTCAATATGAACAGTACCCCA	-	
	4 YEZV F1	2217	GYCCAATTCAAGAATCRCCTA	+	834
	4 YEZV R	3094	ATCTACTAAGCCATTGAGGTTG	-	
	4 YEZV F2	2282	CTTTCCCTRCCCGTAGCWG	+	
	5 YEZV F1	2893	ATCTCTAGGAGGCATATCAGT	+	763
	5 YEZV F2	2956	CCAGATCGCTCTAGTACACA	+	
	5 YEZV R	3699	CTCYGACTCTTTTGGTGCTT	-	
	6 YEZV F1	3535	AACATTRGACAGTCCYGACCGG	+	649
	6 YEZV F2	3607	CACYCTAACTTGCAGCGCT	+	
	6 YEZV R	4237	AGTGGCAGCATACCCCCCTT	-	

Segment	Primer	Position (bp)*	Sequence (5′→3′)	Polarity	Amplicon (bp)
Small					
	1 YEZV F	1	CGTGCTGCGACCCCCCAATAG	+	854
	1 YEZV R2	766	CTTCTTCTGTACAAGGTGC	-	
	1 YEZV R1	840	TCCATCTTCTGCTGGTTGT	-	
	2 YEZV F1	607	GCAGGAACAAGAAGCACTC	+	608
	2 YEZV R	1273	AGCCTTCTTTGATCTTRACA	-	
	2 YEZV F2	684	ATAGGATCTTYAGCCTGCCTTC	+	
	3 YEZV F1	1152	CYAACATGTGTGCAACRGGA	+	464
	3 YEZV R	1676	CCTAGAAGCGGGAAGATGTTGT	-	
	3 YEZV F2	1233	AGATGACTGCWGCCATTGGC	+	

*The position of primers referred to the complete sequences obtained from RNA-seq in this study.

Appendix Table 2. Clinical blood biochemistry test results of the patient at admission

Test	Result	Normal range
Leukocytes count (×10^9 per L)	4.46	4.0–10.0
Lymphocyte cells count (×10^9 per L)	**0.67**	0.90–4.52
Monocytes count (×10^9 per L)	0.39	0.1–0.6
Neutrophils count (×10^9 per L)	3.38	1.55–6.27
Eosinophils count (×10^9 per L)	0.01	0.0–0.76
Basophils count (×10^9 per L)	0.0	0.0–0.1
Lymphocyte percentage (%)	**15.0**	22.6–49.4
Monocytes percentage (%)	8.7	3.0–10.0
Neutrophils percentage (%)	**75.9**	39.0–68.5
Eosinophils percentage (%)	**0.2**	0.4–8.0
Basophils percentage (%)	0.0	0.0–1.0
Red cells count (×10^{12} per L)	5.02	4.30–5.80
Concentration of hemoglobin (g/L)	158.0	131.0–172.0
Platelet count (×10^9 per L)	199	125–350
C-reactive protein (mg/l)	11.1	0.0–3.0
Alanine aminotransferase (U/L)	40	9–50
Aspartate aminotransferase (U/L)	**44**	15–40
Alkaline phosphatase (U/L)	45	45–125
γ -glutamyltransferase	**124**	8–57

*Abnormal values are indicated in bold.

Appendix Table 3. Nucleotide sequence information of the Yezo virus identified in the present study

Strain	Place of sampling in China	Host species	Gene	GenBank Accession no.
T-HLJ01	Heilongjiang	*Ixodes persulcatus*	L	ON563268
T-HLJ01	Heilongjiang	*Ixodes persulcatus*	M	ON563269
T-HLJ01	Heilongjiang	*Ixodes persulcatus*	S	ON563270
T-HLJ02	Heilongjiang	*Ixodes persulcatus*	L	ON563271
T-HLJ02	Heilongjiang	*Ixodes persulcatus*	M	ON563272
T-HLJ02	Heilongjiang	*Ixodes persulcatus*	S	ON563273
T-HLJ03	Heilongjiang	*Ixodes persulcatus*	L	ON563274
T-HLJ03	Heilongjiang	*Ixodes persulcatus*	M	ON563275
T-HLJ03	Heilongjiang	*Ixodes persulcatus*	S	ON563276
T-IM01	Hulunbuir, Inner Mongolia	*Ixodes persulcatus*	L	ON563277
T-IM01	Hulunbuir, Inner Mongolia	*Ixodes persulcatus*	M	ON563278
T-IM01	Hulunbuir, Inner Mongolia	*Ixodes persulcatus*	S	ON563279
T-JL01	Jilin	*Ixodes persulcatus*	L	ON563280
T-JL01	Jilin	*Ixodes persulcatus*	M	ON563281
T-JL01	Jilin	*Ixodes persulcatus*	S	ON563282
H-IM01	Hulunbuir, Inner Mongolia	Human	L	ON563283
H-IM01	Hulunbuir, Inner Mongolia	Human	M	ON563284
H-IM01	Hulunbuir, Inner Mongolia	Human	S	ON563285

Appendix Table 4. Nucleotide sequence identities of large segment (lower left) and amino acid sequence identities of RdRp (upper right) of YEZV*

**Isolate**	T-HLJ01	T-HLJ02	T-HLJ03	T-IM01	T-JL01	H-IM01	HH001–20	HH003–20	HH011–20	SLNV	JANV	SGLV	TCTV1
T-HLJ01	***	99.8	99.8	99.7	99.8	99.8	99.7	99.8	99.7	82.5	45.8	44.9	44
T-HLJ02	98.4	***	99.7	99.7	99.7	100	99.8	99.7	99.7	82.4	45.7	44.8	44
T-HLJ03	98.9	98.4	***	99.6	99.8	99.7	99.6	99.7	99.6	82.4	45.8	44.8	43.9
T-IM01	98.2	98.5	98.3	***	99.6	99.7	99.7	99.7	99.6	82.3	45.8	44.8	44
T-JL01	98.8	98.3	99	98.2	***	99.7	99.6	99.7	99.6	82.4	45.7	44.8	43.9
H-IM01	98.2	98.5	98.3	100	98.3	***	99.8	99.7	99.7	82.4	45.7	44.8	44
HH001–20	97.2	97.4	97.3	97.3	97.2	97.4	***	99.7	99.8	82.5	45.7	44.8	43.9
HH003–20	98.7	98.5	98.8	98.3	98.8	98.3	97.4	***	99.6	82.4	45.7	44.8	43.9
HH011–20	97.3	97.5	97.4	97.4	97.3	97.4	98.4	97.4	***	82.5	45.7	44.8	44
SLNV	70.3	70.1	70.2	70.2	70.2	70.2	70.2	70.1	70.1	***	44.9	43.8	43.7
JANV	51	51.1	50.9	51.1	51	51.1	51.1	51	51.2	51	***	84	63.6
SGLV	50	50	50	50	50.1	50	50.1	50.1	50.1	50.5	72.9	***	63.2
TCTV1	49.5	49.6	49.5	49.6	49.5	49.6	49.6	49.5	49.6	49.4	61.1	61.6	***

*YEZV, Yezo virus; SLNV, Sulina virus strain IxriSL16–03; JANV, Ji'an nariovirus strain NE-JA; SGLV, Songling virus strain HLJ1202; TCTV1, Tacheng tick virus 1 strain TC253. YEZV strains were replaced with the strain name.

Appendix Table 5. Nucleotide sequence identities of small (lower left) and medium (upper right) segments of YEZV*

**Isolate**	T-HLJ01	T-HLJ02	T-HLJ03	T-IM01	T-JL01	H-IM01	HH001–20	HH003–20	HH011–20	SLNV	JANV	SGLV	TCTV1
T-HLJ01	***	98.6	98.8	98.3	98.8	98.3	97.8	98.7	97.4	59.9	43.5	43.4	43.1
T-HLJ02	98.6	***	98.4	98.6	98.4	98.6	97.9	98.5	97.6	59.9	43.5	43.7	43.2
T-HLJ03	98.8	98.4	***	98.2	98.8	98.2	97.7	98.7	97.3	59.9	43.4	43.6	43.2
T-IM01	98.3	98.6	98.2	***	98.2	100	97.8	98.2	97.5	59.7	43.4	43.4	43.3
T-JL01	98.8	98.4	98.8	98.2	***	98.2	97.7	98.6	97.4	59.8	43.4	43.5	43.2
H-IM01	98.3	98.6	98.2	100	98.2	***	97.8	98.2	97.5	59.7	43.4	43.4	43.3
HH001–20	97.8	97.9	97.7	97.8	97.7	97.8	***	97.8	98.6	59.8	43.4	43.8	43.1
HH003–20	98.7	98.5	98.7	98.2	98.6	98.2	97.8	***	97.4	59.7	43.5	43.7	43
HH011–20	97.4	97.6	97.3	97.5	97.4	97.5	98.6	97.4	***	59.8	43.3	43.7	43
SLNV	59.9	59.9	59.9	59.7	59.8	59.7	59.8	59.7	59.8	***	40.5	41	40.6
JANV	43.5	43.5	43.4	43.4	43.4	43.4	43.4	43.5	43.3	40.5	***	70.9	54.6
SGLV	43.4	43.7	43.6	43.4	43.5	43.4	43.8	43.7	43.7	41	70.9	***	55.1
TCTV1	43.1	43.2	43.2	43.3	43.2	43.3	43.1	43	43	40.6	54.6	55.1	***

*YEZV, Yezo virus; SLNV, Sulina virus strain IxriSL16–03; JANV, Ji'an nariovirus strain NE-JA; SGLV, Songling virus strain HLJ1202; TCTV1, Tacheng tick virus 1 strain TC253. YEZV strains were replaced with the strain name.

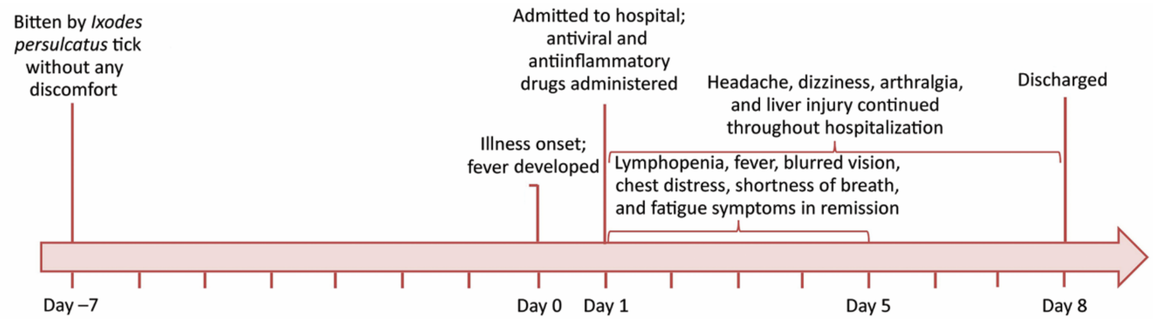

Appendix Figure 1. Schematic organization of Yezo virus (YEZV) genome. The genome is composed of large (L), medium (M), and small (S) RNA segments, and the predicted open reading frames are shown in boxes. GPC, glycoprotein precursor; N, nucleocapsid; RdRp, RNA-dependent RNA polymerase.

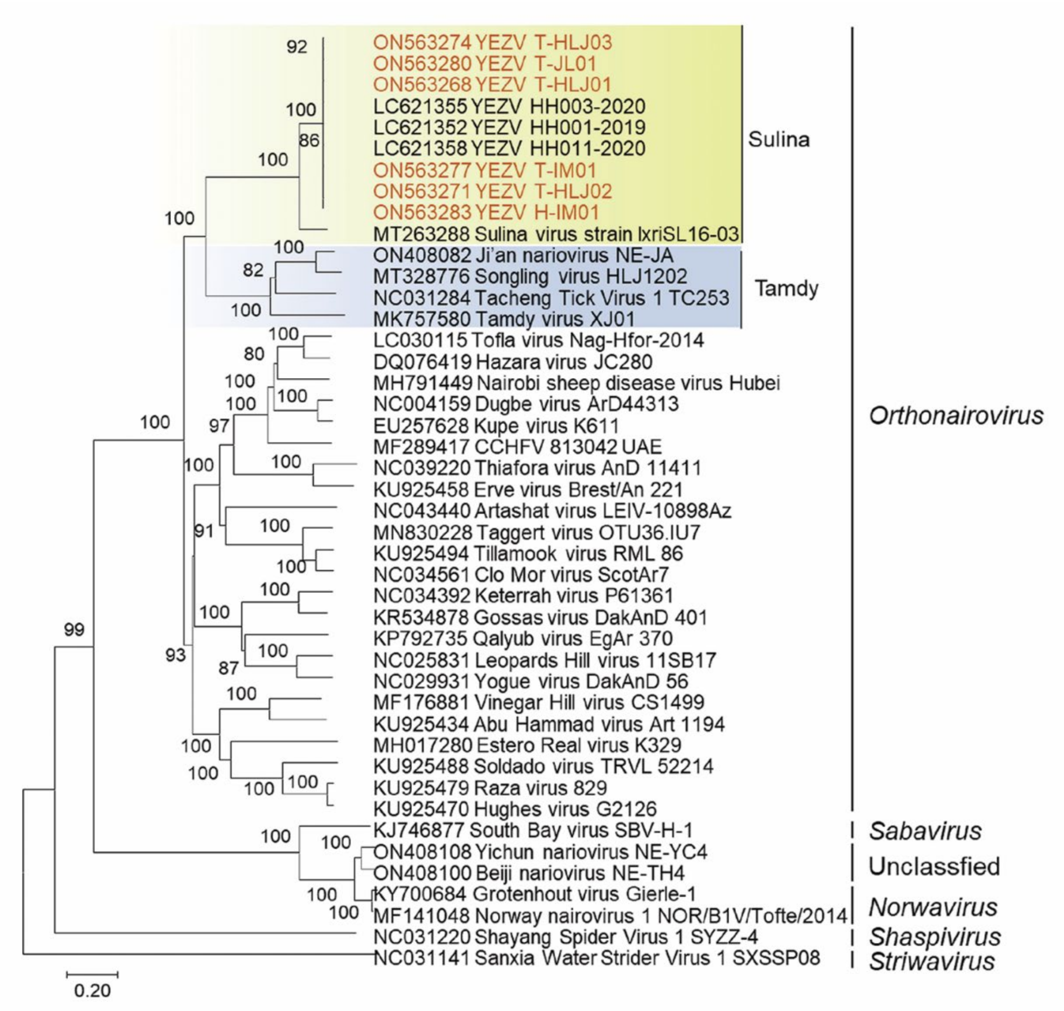

Appendix Figure 2. Phylogenetic analyses of Yezo virus (YEZV). A) Amino acid sequences of glycoprotein protein of family *Nairoviridae* including YEZVs. B) Amino acid sequences of nucleocapsid protein of family *Nairoviridae* including YEZVs. Amino acid sequences of representative viral strains were downloaded from NCBI public databases and aligned together by using MEGA version 7.0. Numbers along branches are bootstrap values. Bootstrapping analyses of 1,000 replicates were conducted, and values >70 were considered significant and are shown in the trees. Scale bars indicate amino acid substitutions per site.
